# Indications for increase in caesarean delivery

**DOI:** 10.1186/s12978-019-0723-8

**Published:** 2019-05-30

**Authors:** Paula da Silva Charvalho, Mira Hansson Bittár, Ylva Vladic Stjernholm

**Affiliations:** 1 Obstetric Unit, Department of Women’s and Children’s Health, Karolinska University Hospital and Karolinska Institutet, SE-171 76 Stockholm, Sweden; 2 Educational Programme in Medicine, Karolinska Institutet, SE-171 76 Stockholm, Sweden

## Abstract

**Background:**

The increasing caesarean delivery rate worldwide is followed by increased maternal morbidity due to pathological placentation, peripartum hysterectomy and obstetric bleeding. The aim of this study was to investigate the indications for caesarean delivery.

**Study design:**

A retrospective observational study. Data were retrieved from the Swedish Pregnancy Register and obstetric records at a tertiary hospital in Sweden between the early 1990s and 2015.

**Results:**

Caesarean delivery in Sweden increased from 10% in the early 1990s to 17% in 2015 concomitantly with decreased instrumental delivery and increased labour induction. Most planned caesareans at the tertiary hospital were performed on maternal request with a rate increasing from 0.6 to 4.6% of all deliveries (*p* < 0.001), and 60% of these women reported secondary fear of vaginal delivery. The second most common indication previous uterine scar increased from 1.2 to 2.3% (*p* < 0.001). Most urgent caesareans in 2015 were carried out because of prolonged labour with the rate increasing from 2.1% to 5.4% of all deliveries (*p* < 0.001). The second most common indication was imminent fetal asphyxia which increased from 2.4 to 2.6% (*p* < 0.01).

**Conclusions:**

The Swedish caesarean delivery rate increased concomitantly with a decrease in instrumental delivery and an increase in labour induction. Most of the planned caesareans were performed on maternal request and most of the urgent caesareans were carried out because of prolonged labour. These findings emphasise the importance of standardised definitions of maternal request and follow-up after a negative birth experience, as well as adequate definitions of prolonged labour and foetal asphyxia to decrease unnecessary caesareans.

## Plain English summary

Improved maternal health is one of the United Nations’ Millennium Development Goals. The increasing caesarean delivery rate worldwide is accompanied by reports on maternal morbidity due to pathological placentation, massive obstetric bleeding and peripartum hysterectomy. According to the World Health Organization caesareans rates higher than 10% at a population level are not associated with reduction in maternal and newborn mortality rates. Caesareans on maternal request are reported in rates of 1–48% of all caesareans in public sector units and up to 60% of all caesareans in the private sector particularly in high-income urban areas.

The aim of this study was to investigate the indications for increased caesarean delivery. Data were retrieved from the Swedish Pregnancy Register and from obstetric records at a tertiary hospital in Sweden between the early 1990s and 2015.

We found, that the national caesarean delivery rate increased concomitantly with an increase in labour induction and a decrease in instrumental delivery. Planned caesareans on maternal request was the primary indication at the tertiary hospital in 2015, and secondary fear of vaginal delivery after a negative birth experience was reported by a majority of these women. The second most common indication was previous uterine scar. Most of the urgent caesareans were carried out because of prolonged labour, followed by imminent fetal asphyxia.

In conclusion, these findings emphasise the importance of standardised definitions of ‘maternal request’ and fear of vaginal delivery as well as systematic follow-up after a negative birth experience in order to decrease unnecessary caesareans.

## Background

Improved maternal health is one of the United Nations’ Millennium Development Goals. According to data from 150 countries, the worldwide caesarean section (CS) rate increased progressively from 7% in 1990 to 19% in 2014 in both developed and developing countries [1, 2]. Latin America and the Caribbean region reported the highest CS rate 42%, followed by North America 32%, Oceania 31%, Europe 25%, Asia 19% and Africa 7% [1]. The rising caesarean delivery rate is accompanied by reports on increasing maternal morbidity due to pathological placentation, peripartum hysterectomy and massive obstetric bleeding [1, 2].

The extent to which caesareans on maternal request has contributed to this increase has long been a matter of debate. Caesareans on maternal request are reported in rates of 1–48% of caesareans in the public sector and 60% in the private sector [3–5]. Controversy exists regarding the definition of ‘maternal request’, the differences in hospitals’ and obstetricians’ attitude to perform a CS on maternal request in the absence of medical indication, and the high rate of such operations in high-income urban areas and private hospitals as compared to rural areas and public health-care systems [3–5]. In contrast, more than 90% of pregnant women claim that they want to give birth in a natural way, according to a Swedish study [6].

The aim of this study was to investigate the indications for caesarean delivery between the early 1990s and 2015 to get an overview of the increasing trend.

## Material and methods

Ethics approval was obtained from the Ethics Board for Medical Sciences in Stockholm April 92,015, No 2014/255–31. Data on mode of delivery in Sweden between the early 1990s and 2015 were retrieved from the Swedish Medical Birth Register which contains data on 97% of deliveries in Sweden based on the World Health Organization (WHO) International Classification of Diseases (ICD)-10 and delivery charts [7]. Approximately 20,000 caesareans were performed in Sweden in 2015. As information about indications for caesarean delivery is not provided by the register, we collected such data from obstetric records at a tertiary hospital in Sweden [8]. Approximately 1700 caesareans, that is 9% of caesareans in Sweden, were carried out at the Karolinska University Hospital in 2015. We choose to investigate original obstetric records in order to avoid incomplete or misleading information based on ICD-10 only. Obstetric records were reviewed independently by two investigators and a subset of cases was reviewed repeatedly to assure accuracy. This study was initiated as a quality control project and a Medical Degree Project and included all caesareans during the years studied. Women with intrauterine foetal death and lethal foetal malformations were excluded. Based on previous results [8], we assumed that CS on maternal request would increase from 10% of planned CS in the early 1990s to 25% in 2015. We calculated that 97 participants in each group would be needed when aiming at a significance level of 5 and 80% power. A *p* value < 0.05 was considered significant. Since all data were anonymised and compiled on a group basis only a statement on individual consent was not required.

The indications for planned CS included the group of maternal request (psychosocial indication, non-medical indication), which was fear of vaginal delivery or maternal request without any co-existing medical indication at a normal gestational age. Fear of vaginal delivery was assessed by a midwife followed by an obstetrician or a psychotherapist. The group of previous uterine scar included women with two or more caesareans, a transmural corporal incision or pathological placentation (placenta praevia and/or placenta accreta). One previous CS is not an indication for planned CS in Sweden, and women requesting a CS after one previous CS were therefore referred to the group maternal request. The group of maternal intercurrent disease included women with severe cardiovascular disease, inflammatory bowel disease, malignancy etc. The group of previous sphincter injury was women with a third or fourth-degree perineal laceration, complicated by a re-operation or persistent sequelae. The group of foetal reason was pregnancy with estimated foetal weight > 4500 g, severe foetal disease or malformation. The group narrow pelvis included women with a pelvic outlet index < 29.5 cm (the sum of sagittal pelvic outlet diameter, interspinal diameter and transverse diameter), an interspinal diameter of < 8.0 cm, or a pelvic inlet sagittal diameter of < 10 cm according to pelvic x-ray. Women with a previous CS in combination with a pelvic outlet index between 29.5–30.5 cm were also included in this group. The group of duplex pregnancy included women with twin pregnancy with the first twin in a breech presentation.

Urgent CS was carried out within 30 min–8 h, and immediate CS within 15 min because of an immediate threat to maternal or foetal health. The group of prolonged labour was a failure to progress for more than 3–4 h during the first stage of labour or more than 2–3 h during the second stage. The group of imminent foetal asphyxia was a pathological cardiotocography (CTG) registration or a pathological scalp-lactate sample > 4.8 mmol/L. The group pregnancy complication included women with severe preeclampsia, diabetes mellitus with complications, Rhesus immunisation and other severe complications.

Continuous data were analysed with one-way analysis of variance (ANOVA) and categorical data with Mann-Whitney U test. Statistical significance was set at a two tailed *p*-value of < 0.05.

## Results

**Fig. 1:**
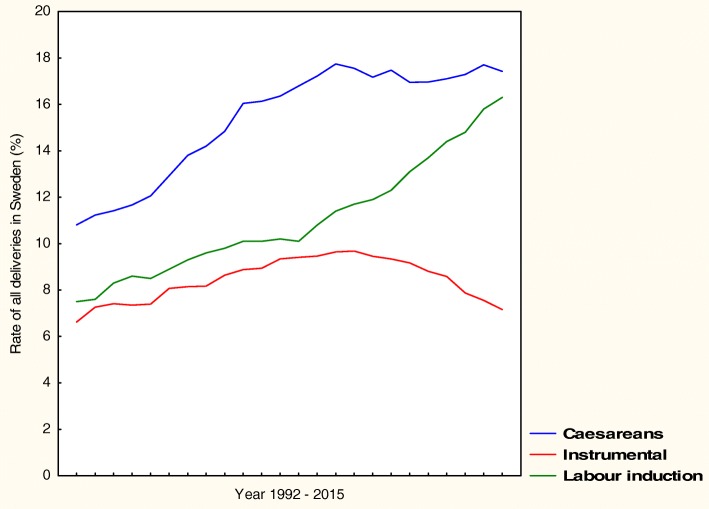
Mode of delivery in Sweden 1992–2015. The Swedish Medical Birth Register

The CS rate in Sweden increased from 10% in the early 1990s to 17% in 2015, concomitantly with increased labour induction and decreased instrumental delivery, as shown in Fig. 1. The mean age among delivering women in Sweden increased from 28 to 30 years and mean BMI was 25 [7, 9].

**Fig. 2:**
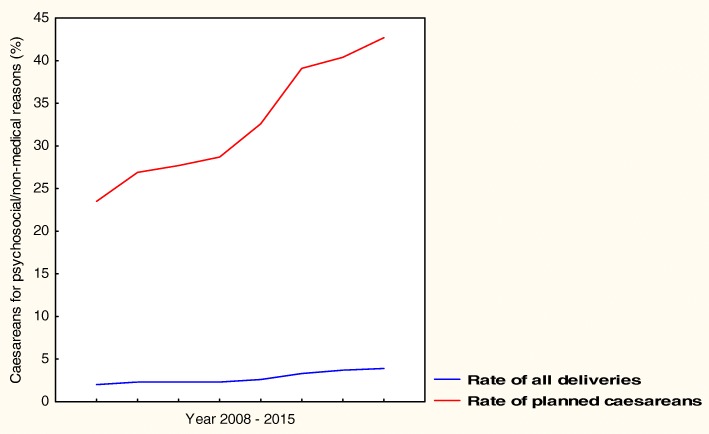
Planned caesareans on maternal request 2008–2015. ICD-10 Karolinska University Hospital

The CS rate at the tertiary hospital increased from 11% in the early 1990s to 22% in 2015, as shown in Table 1. Most of the planned caesareans (76%) were carried out on maternal request, followed by the groups previous uterine scar and breech presentation, as shown in Table 2. The rate of planned caesarean on maternal request increased from 0.6 to 4.6% of all deliveries (*p* < 0.001). Quantification of this indication using ICD-10 became possible in 2008, and the progressive trend is shown in Fig. 2. In 2015, 77% of the women who underwent planned CS on maternal request were parous, 57% had a previous CS, and 20% had a previous vaginal delivery. Secondary fear of vaginal delivery after a negative birth experience was reported by 60% (2.8% of delivering women), primary fear of vaginal delivery by 34% (1.5%), whereas 5% (0.2%) was related to a pre-existing psychiatric health disorder such as severe depression, bipolar disease or an attention deficit disorder, and 1% (0.04%) was carried out on maternal request without further explanation. Planned CS because of previous uterine scar increased from 1.2 to 2.3% of all deliveries (*p* < 0.001), and planned CS because of breech presentation decreased from 1.6 to 1.5% (p < 0.001). The group of planned caesarean because of previous sphincter injury increased from 0.1 to 0.8% (p < 0.001) and the group of foetal reason from 0.1 to 0.8% (p < 0.001). The low rates of planned CS because of maternal intercurrent disease, narrow pelvis and duplex pregnancy (data not shown) were unchanged.

**Table 1:** Mode of delivery

Year	1992	2005	2015
	n (%)	n (%)	n (%)
Sweden			
Deliveries	121,123	99,361	114,981
Caesarean	(10.8)	(17.2)	(17.4)
Karolinska University Hospital			
Deliveries1	(7.4)	(9.9)	7831 (7.9)
Labour induction	(6.0)	(17.0)	1730 (22.1)
Instrumental delivery	(7.7)	(13.0)	593 (7.6)
Caesarean	(11.4)	(19.9)	1722 (21.7)

Source: The Swedish Pregnancy Register and ICD-10 Karolinska University Hospital

Unfortunately information about rates only was available for the years 1992 and 2005

^1^Rate of deliveries in Sweden

**Table 2:** Indications for planned caesarean

	Rate of all deliveries					Rate of all planned caesareans		
Year	1992 n (%)	2005 n (%)	2015 n (%)	*p* value 1992/2015	*p* value 2005/2015	1992 (%)	2005 (%)	2015 (%)
Maternal request	51 (0.6)	380 (3.9)	362 (4.6)	< 0.001	< 0.001	(10.5)	(38.5)	(41.2)
Previous uterine scar	111 (1.2)	158 (1.6)	180 (2.3)	< 0.001	< 0.001	(22.8)	(16.0)	(20.5)
Breech presentation	140 (1.6)	211 (2.1)	116 (1.5)	< 0.001	< 0.001	(28.8)	(21.4)	(13.2)
Previous sphincter injury	7 (0.1)	72 (0.7)	63 (0.8)	< 0.001	< 0.05	(1.4)	(7.3)	(7.2)
Foetal reason	7 (0.1)	31 (0.3)	62 (0.8)	< 0.001	< 0.001	(1.4)	(3.1)	(7.0)

Source: Obstetric records Karolinska University Hospital

Most the urgent caesareans (90%) in 2015 were carried out because of prolonged labour, followed by imminent foetal asphyxia and preterm caesarean, as shown in Table 3. Immediate caesareans were 8% of non-planned caesareans (0.9% of all deliveries). Urgent CS because of prolonged labour increased from 2.1 to 5.4% of all deliveries (*p* < 0.001) between the early 1990s and 2015, and was related to foetal malpresentations such as occipital posterior presentation or asynclitism in 15%. Urgent CS because of imminent foetal asphyxia increased from 2.4 to 2.6% between the early 1990s and 2015 (*p* < 0.01), and urgent preterm caesarean increased from 0.2 to 1.6% (p < 0.001). The low rates of urgent CS because of pregnancy complication or uterine rupture were unchanged.

**Table 3:** Indications for urgent caesarean

	Rate of all deliveries					Rate of all urgent caesareans		
Year	1992 n (%)	2005 n (%)	2015 n (%)	*p* value 1992/2015	*p* value 2005/2015	1992 (%)	2005 (%)	2015 (%)
Prolonged labour	192 (2.1)	449 (4.6)	432 (5.4)	< 0.001	< 0.001	(37.0)	(46.1)	(51.2)
Imminent foetal asphyxia	218 (2.4)	435 (4.4)	203 (2.6)	< 0.01	< 0.001	(42.0)	(44.7)	(23.1)
Preterm caesarean	19 (0.2)	12 (0.1)	130 (1.6)	< 0.001	< 0.001	(3.7)	(1.2)	(15.4)
Pregnancy complication	85 (0.9)	76 (0.8)	75 (0.9)	> 0.05	> 0.05	(16.4)	(7.8)	(8.9)
Uterine rupture	5 (0.1)	2 (0.0)	3 (0.0)	> 0.05	> 0.05	(0.9)	(0.2)	(0.3)

Source: Obstetric records Karolinska University Hospital

## Discussion

The main findings in this study were that the CS rate in Sweden increased from 10% in the early 1990s to 17% in 2015 concomitantly with an increase in labour induction and a decrease in instrumental delivery. According to the WHO, caesareans are effective in saving maternal and infant lives only when they are required for medically indicated reasons and CS rates higher than 10% at a population level are not associated with reduction in maternal and newborn mortality rates [2]. The Robson classification system has been suggested by the WHO as a global standard for assessing and comparing CS rates between healthcare facilities [2]. The dominant Robson groups of CS in Sweden and at the tertiary hospital in 2015 were Group 2, primiparous women with single cephalic pregnancy 37 weeks or more, who either had labour induced or were delivered by CS before labour and Group 5, multiparous women with single cephalic pregnancy 37 weeks or more and at least one previous uterine scar [9, 10].

At the tertiary hospital planned CS on maternal request increased progressively between the early 1990s and 2015 and was the primary indication for a planned caesarean in 2015, followed by the groups previous uterine scar and breech presentation. Secondary fear of vaginal delivery after a negative birth experience was reported by a majority of these women, which emphasises the importance of a positive first birth experience and structured follow-up after traumatic childbirth [6, 11, 12]. Fear of vaginal delivery, which is related to pre-existing psychosocial burdens such as anxiety, depression, abuse, and violence has been estimated in 5─6% of pregnant women and in 11% if negative expectations are included in the definition [6, 7, 11]. According to a Norwegian study, 80% of women who experience obstetric complications neither consider the birth a negative overall experience nor develop a fear of vaginal delivery [11]. The discrimination between fear and physiological anxiety can be difficult since pregnancy-related anxiety is common and increasing towards parturition. Therefore, standardised definitions of maternal request and fear of vaginal delivery have been warranted [3, 5, 13]. In Sweden 2015, 8% of pregnant women received extended support because of fear of vaginal delivery [9]. Extended support should include repeated meetings with a psychosocial team and objective information about benefits and risks related to different delivery modes on future reproductive health [2, 5, 6, 11, 12]. It has been reported previously, that the attitudes among midwives and obstetricians influence patient’s choice [3, 5, 8, 14]. Thus, a ‘coping attitude’ rather than an ‘autonomy attitude’ is strongly associated with a change in desire for CS [14]. The increasing CS rate because of previous uterine scar was recognised as a consequence of the rising CS rate. As the risk of pathological placentation increases with the number of previous caesareans, women with pathological placentation and repeated CS were referred to the group previous uterine scar [15]. Caesareans because of breech presentation decreased when external versions were carried out at an earlier gestational age, which improved the success rate. The increased CS rate due to previous sphincter injury is a consequence of improved diagnostics and the delivery technique, and stresses the importance of routine perineal protection at delivery. The increased rate of CS for foetal reasons was recognised as a result of improved ultrasound diagnostics.

Urgent CS because of prolonged labour increased at the tertiary hospital between the early 1990s and 2015. Insufficient support during delivery, high maternal age and BMI have been reported as risk factors of prolonged labour [16–18]. However, mean age and BMI among women who delivered by urgent CS because of prolonged labour did not differ from that of all delivering women. According to a recent WHO report, normal progression of the first stage of spontaneous labour appear to be slower than the previously assumed 1 cm/hour regardless of parity. Interventions to expedit labour to reach a cervical dilatation 1 cm/hour may therefore be inappropriate, especially during the early opening stage in primiparous and multiparous women [19].

Instrumental delivery decreased in Sweden and at the tertiary hospital. Approximately 99% of instrumental deliveries in Sweden are carried out using vacuum extraction. Instrumental delivery requires team collaboration and practical training [20] and inadequate training could result in a tendency to choose a CS instead of an instrumental delivery in an urgent situation, which might explain the declining instrumental delivery rate observed here, as reported from other obstetric units [21]. Induced labour increased, and was at our hospital mostly planned because of postterm pregnancy, prelabour rupture of foetal membranes or hypertensive disease. In contrast to others, we found induced labour to be a risk factor for urgent CS [21, 22]. Trial of vaginal delivery at our hospital was followed by urgent CS in 14% and induced labour in 30% [22]. We assume, that urgent CS because of imminent foetal asphyxia decreased as a result of staff education on foetal monitoring [23]. Neonatal asphyxia according to Apgar scores and umbilical blood gas values (data not shown) did not increase.

The categorisation of indications for CS were unchanged between the years studied, except for the group urgent preterm CS, which included women with threatening preterm birth in combination with signs of urgent foetal asphyxia and/or a breech presentation between 28 + 0–36 + 6 weeks in 1992, between 25 + 0–36 + 6 weeks in 2005, and between 23 + 0–36 + 6 weeks in 2015. Urgent preterm CS increased after altered guidelines recommending referral of women with threatening preterm birth to a tertiary hospital and active management including CS at an earlier gestational age. This development warrants long-term follow-up of maternal and child health since preterm CS between 24─33 weeks reduces neonatal mortality and morbidity only when performed because of fetal distress or breech presentation [24]. Also, preterm CS more often requires a high transmural corporal incision than term CS, due to an inadequately developed lower uterine segment in preterm gestation. As a result, preterm CS increases the risk of pathological placentation and uterine rupture in subsequent pregnancies more than term CS [25].

Strengths in this study were the high number of observations retrieved from national register data [7], and the high accuracy data obtained from obstetric records. A limitation was that data from obstetric records represented only 9% of caesareans in Sweden.

## Conclusions

In conclusion, the caesarean delivery rate in Sweden increased concomitantly with an increase in labour induction and a decrease in instrumental delivery. Most of the planned caesareans at the tertiary hospital were performed on maternal request, followed by previous uterine scar and breech presentation. Most of the urgent caesareans were carried out because of prolonged labour, followed by imminent foetal asphyxia and preterm caesarean. These findings emphasise the importance of standardised definitions of ‘maternal request’, structured follow-up after a negative birth experience, as well as adequate definitions of prolonged labour and foetal asphyxia in order to decrease unnecessary caesareans.

## Abbreviations

CS: Caesarean section
CTG: Cardiotochography
ICD-10: International Classification of Diseases
WHO: World Health Organization

## References

[1] Betrán AP, Ye J, Moller AB, Zhang J, Gülmezoglu AM, Torloni MR (2016). The increasing trend in cesarean section rates: global, regional and national estimates: 1990-2014. PLoS One.

[2] World Health Organization. WHO statement on cesarean section rates. Geneva, Switzerland 2015. Available at http://www.who.int.

[3] Habiba M, Kaminski M, Da Fré M, Marsal K, Bleker O, Librero J (2006). Caesarean section on request: a comparison of obstetricians’ attitudes in eight European countries. Br J Obstet Gynecol.

[4] Barber E, Lundsberg LS, Bolanger K, Pettker CM, Funai EF, Illuzi JL (2011). Indications contributing to the increasing cesarean delivery rate. Obstet Gynecol.

[5] Lavender T, Hofmeyr GJ, Neilson JP, Kingdon C, Gyte GML (2012). Cesarean section for non-medical reasons at term. Cochrane Database Syst Rev.

[6] Waldenström U, Hildingsson I, Ryding EL (2006). Antenatal fear of childbirth and its association with subsequent caesarean section and experience of childbirth. Br J Obstet Gynaecol.

[7] The Swedish National Board of Health and Welfare. The Medical Birth Register. Statistics on pregnancies, deliveries and newborn babies. Available at http://www.socialstyrelsen.se

[8] Vladic Stjernholm Y, Pettersson K, Eneroth E (2010). Changed indications for cesarean sections. Acta Obstet Gynecol Scand.

[9] The Swedish pregnancy register 2015. Available at https://www.graviditetsregistret/.se

[10] Torloni M, Betrán A, Souza J, Widmer M, Allen T, Gulmezoglu M (2011). Classifications for cesarean section: a systematic review. PLoS One.

[11] Storksen HT, Gartus-Niegel S, Vangen S, Eberhard-Gran M (2012). The impact of previous birth experiences on maternal fear of childbirth. Acta Obstet Gynecol Scand.

[12] Bastos MH, Furuta M, Small R, McKenzie-McHarg K, Bick D. Debriefing interventions for the prevention of psychological trauma in women following childbirth. Cochrane Database Syst Rev 2015, Issue 4. Art. No.: CD007194. DOI: 10.1002/14651858.CD007194.pub2. 10.1002/14651858.CD007194.pub2 PMC11452364 25858181

[13] Brunton RJ, Dryer R, Saliba A, Kohlhoff J (2015). Pregnancy anxiety: a systematic review of current scales. J Affect Dis.

[14] Halvorsen L, Nerum H, Sorlie T, Oian P (2010). Does counselor’s attitude influence change in a request for a caesarean in women with fear of birth?. Midwifery.

[15] Silver RM (2015). Abnormal placentation: Placenta previa, vasa previa, and placenta accreta. Obstet Gynecol.

[16] Hodnett ED, Gates S, Hofmeyr GJ, Sakala C. Continuous support for women during childbirth. Cochrane Database Syst Rev. 2012;(10):CD003766. 10.1002/14651858.CD003766.pub4 PMC4175537 23076901

[17] Weiss JL, Malone FD, Emig D, Ball RH, Nyberg DA, Comstock CH (2004). Obesity, obstetric complications and cesarean delivery rate – a population-based screening study. Am J Obstet Gynecol.

[18] Smith GC, Cordeaux Y, White IR, Pasupathy D, Missfelder-Lobos H, Pell JP (2008). The effect of delaying childbirth on primary cesarean section rates. PLoS Med.

[19] Oladapo OT, Souza JP, Fawole B, Mugerva K, Pedoná G, Alves D (2018). Progression of the first stage of spontaneous labour: a prospective cohort study in two sub-Saharan African countries. PLoS Med.

[20] Draycott T, Sibanda T, Owen L, Akande V, Winter C, Reading S, Whitelaw A (2006). Does training in obstetrics emergencies improve neonatal outcome?. Br J Obstet Gynaecol.

[21] Caughey AB, Cahill AG, Guise JM, Rouse DJ (2014). American College of Obstetricians and Gynecologists. Society for Maternal-Fetal Medicine. Safe prevention of the primary cesarean delivery. Am J Obstet Gynecol.

[22] Thorbiörnson A, Vladic T, Vladic Stjernholm Y (2016). Oral versus vaginal prostaglandin for labor induction. J Matern Fetal Neonatal Med.

[23] Holzmann M, Wretler S, Cnattingius S, Nordström L (2015). Cardiotocographic patterns and risk of intrapartum acidemia. J Perinat Med.

[24] Reddy U, Zhang J, Sun L, Chen Z, Raju T, Laughon K (2012). Neonatal mortality by attempted route of delivery in early preterm birth. Am J Obstet Gynecol.

[25] Lannon SM, Guthrie KA, Vanderhoeven JP, Gammill HS (2015). Uterine rupture risk after periviable cesarean delivery. Obstet Gynecol.

